# Amplicon-based analysis reveals link between adolescent acne and altered facial skin microbiome induced by negative emotional states

**DOI:** 10.3389/fcimb.2025.1543616

**Published:** 2025-03-19

**Authors:** Yu Chen, Lixia Peng, Yueying Li, Yusheng Peng, Siqi Dai, Kai Han, Jinge Xin

**Affiliations:** ^1^ Department of Dermatology, The People’s Hospital of Baiyun District, Guangzhou, China; ^2^ Department of Dermatology, Nanfang Hospital Taihe Branch, Guangzhou, China; ^3^ Department of Dermatology, Nanfang Hospital, Southern Medical University, Guangzhou, China

**Keywords:** skin microbiome, acne vulgaris, adolescents, negative emotions, 16S rRNA 43 sequencing, bioinformatics

## Abstract

**Introduction:**

The skin microbiome is integral to maintaining skin homeostasis and is involved in the pathogenesis of acne. Emerging evidence supporting the ‘brain-skin axis’ suggests that psychological stress may exacerbate acne. Both negative emotional states and acne are highly prevalent among adolescents. Although research has begun to explore this relationship, the role of the skin microbiome in adolescents experiencing emotional disturbances and acne remains poorly understood.

**Methods:**

166 adolescents aged 15–18 were divided into four distinct groups based on their emotional health and acne severity: no acne or negative emotions (NC), acne without negative emotions (NS), negative emotions without acne (YC), and acne with negative emotions (YS). Skin samples were collected from each participant’s forehead and analyzed using high-throughput sequencing techniques, followed by comprehensive bioinformatics analyses to evaluate the microbial composition and diversity across the different groups.

**Results:**

Adolescents with both acne and negative emotions exhibited significantly higher acne severity (IGA 2.675 ± 0.090) compared to the group with acne but without negative emotions (IGA 1.952 ± 0.136). Distinct microbial community patterns emerged among the groups, with acne-affected individuals displaying increased α-diversity. Additionally, negative emotions were associated with heightened β-diversity differences between acne-affected individuals. The predominant bacterial phyla identified were Firmicutes, Bacteroidetes, Proteobacteria, and Fusobacteria, with *Acinetobacter* being more abundant, and *Roseomonas* and *Cutibacterium* being less prevalent in adolescents experiencing negative emotions.

**Conclusion:**

This study revealed that the bacterial biomarkers of the disease change when acne is accompanied by negative emotions. *Cutibacterium*, *Acinetobacter*, and *Roseomonas* may be key contributors to acne exacerbation. These findings underscore the importance of considering both emotional and microbiological factors in the management of adolescent acne, particularly within the context of the brain-skin connection.

## 1 Introduction

The skin microbiome is a highly complex and dynamic ecosystem, comprising approximately one million microorganisms per square centimeter across the 1.8 m² surface of human skin (Belkaid and Naik, 2013; Belkaid and Segre, 2014; Schommer and Gallo, 2013). Skin microbial communities play essential roles in sustaining skin homeostasis by reinforcing the skin barrier (Almoughrabie et al., 2023), maintaining pH balance (Nakamura et al., 2020), inhibiting colonization by pathogenic bacteria (Claesen et al., 2020; Nagy et al., 2006), and modulating both innate and adaptive immunity (Agak et al., 2018; Sanford et al., 2016). The skin microbiome is involved in the pathogenesis of various dermatological conditions, including acne (Linehan et al., 2018; Naik et al., 2015, 2012; Scharschmidt et al., 2015; Weckel et al., 2023). Recent research has highlighted the “brain-skin connection”, which investigates the interplay between neurological and dermatological health (Marek-Jozefowicz et al., 2022). Stress-induced hormonal fluctuations can disrupt the central clock of the brain, which is crucial for maintaining skin physiology (Mortimer et al., 2024). These changes can reduce skin hydration, decrease blood flow and lipid production, and alter the physiological environment of the skin, thereby affecting the composition of the skin microbiome (Costello et al., 2009; Dhabhar, 2000; Grice et al., 2009; Grice and Segre, 2011; McGinley et al., 1980). Notably, mental health disorders and skin conditions often manifest concurrently with depression and anxiety frequently accompanying by acne (Costello et al., 2009). Psychological stress has also been identified as a factor that can worsen acne severity (Loney et al., 2008; Uhlenhake et al., 2010).

In 2024, approximately 1.2 billion of the world’s population will be adolescents, accounting for one-sixth of the global population (Trask, 2024). Adolescents are in a transition period from childhood to adulthood and experience significant physiological and psychological changes, including hormonal shifts and emotional development (Eiland and Romeo, 2013). In this population, negative emotions such as depression and anxiety, as well as dermatological issues such as acne, are prevalent. According to the World Health Organization, one in seven adolescents aged 10–19 years is affected by negative emotions, accounting for 13% of the global disease burden in this demographic (Trask, 2024). Acne affects nearly 90% of adolescents worldwide, making it one of the most widespread conditions with an estimated annual economic impact of $4 billion annually (Tan and Bhate, 2015). Given the distinct age-related variations in the skin microbiome (Luna, 2020), research into the brain-skin axis in adolescents is essential for developing strategies for managing acne that is exacerbated by psychological stress.

We performed a cross-sectional pilot analysis of adolescents aged 15–18, using 16S rRNA sequencing of the V3-V4 region to investigate the diversity and composition of the facial microbiome in individuals with acne and mood disorders. A machine learning approach was utilized to identify significant microbial biomarkers and microbiome function was predicted using PICRUSt2. The aim was to characterize the microbiological characteristics of acne in adolescents with negative emotions, potentially guiding the development of innovative treatment strategies for this population.

## 2 Materials and methods

### 2.1 Subjects and sample acquisition

**Figure 1:**
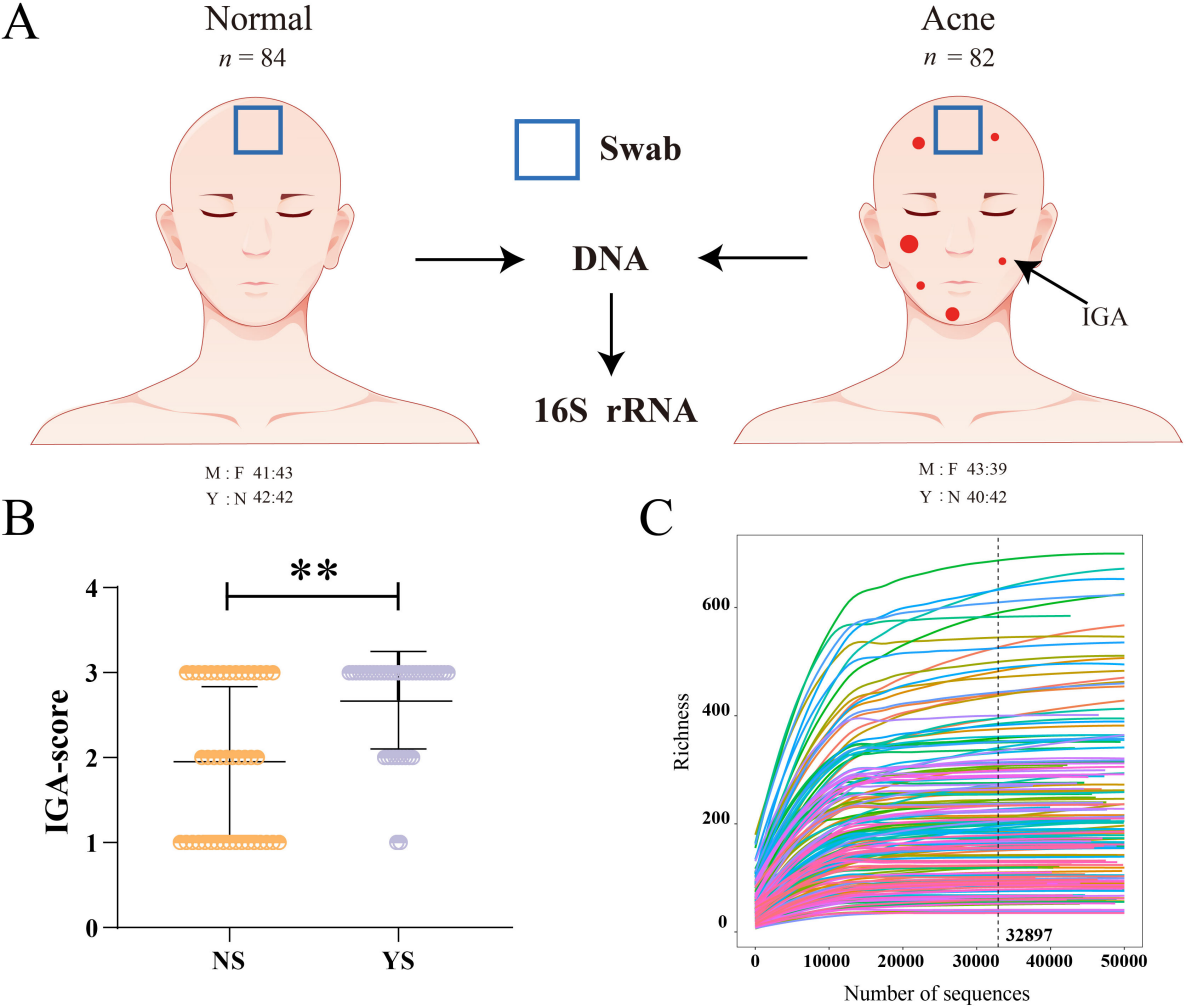
Experimental design and subject demographics. **(A)** Schematic of cohort characteristics and skin site location of skin microbiota collection. **(B)** IGA score for each subject with acne, ** indicates p<0.01. **(C)** The rarefaction curves tend to attain the saturation plateau showing that the skin microbiota of all samples was large enough to estimate the phenotype richness and microbial community diversity. M, male; F, female; A, acne; N, normal; Y, negative emotions; N, no negative emotions; IGA, investigator global assessment of acne disease.

A total of 166 volunteers, including males and females, ages 15-18 years, with and without acne, were enrolled after providing informed assent with written informed parental consent. During the study visit, dermatologists assessed acne severity using the 5-point investigator global assessment (IGA) scale (Schneider et al., 2023; **Figure 1A**; **Supplementary Table S1**). The inclusion criteria were as follows: 1) subjects who had lived in Guangzhou, Guangdong Province, China, for more than two years. 2) Healthy subjects with no underlying skin/medical conditions other than acne, if present. 3) No history of oral antibiotic dermabrasion or facial laser therapy within 2 months prior to the start of the study. 4) Subjects had not used topical antibiotics, benzoyl peroxide, or salicylic washes within one month prior to the start of the study. 5) Subjects had not used any cleansing or skincare products on their faces within 12 h prior to the start of the study. 6) The female participants were not breastfeeding, pregnant, or menstruating. With reference to previous studies, skin swab was collected from forehead area (Schneider et al., 2023). For subjects meeting the inclusion criteria, a 7 cm^2^ area in the center of the forehead was swabbed at medium pressure with a pre-wet sterile synthetic cotton swab for 30 s and stored at -80°C until sequencing. Finally, swab samples were collected from 166 volunteers. All acne patients have acne on the forehead.

To assess the psychological burden on all participants, two standardized and validated questionnaires for depression and anxiety were administered: the Self-Rating Anxiety Scale (SAS) (Zung, 1971) and the Self-Rating Depression Scale (SDS) (Zung, 1965). Each questionnaire contained 20 questions. According to the primary screening diagnostic criteria of Chinese anxiety and depression norms: SAS ≥50 and SDS ≥53 represent diagnosable anxiety and depression, respectively (Wang et al., 2021). Demographic and clinical data are provided in **Supplementary Table S2**.

### 2.2 DNA extraction, 16S rRNA gene amplification and sequencing

DNA was extracted immediately from the collected frozen swabs using commercial DNA kit (Nanjing Vazyme Biotech Co., Ltd., Nanjing, China) and following the manufacturer’s instructions. PCR amplification of the V3-V4 region of 16S rRNA was using the primers: 338F (5’-ACTCCTACGGGAGGCAGCA-3’) and 806R (5’ GGACTACHVGGGTWTCTAAT-3’). Paired-end 2 × 250 bp sequencing of the bacterial 16S rRNA gene was performed using the Illumina MiSeq sequencing platform. The reaction of PCR was carried out in the 25 μL system that contained Buffer (5×, 5 µl), Fast pfu DNA Polymerase (5 U/µl, 0.25 µl), dNTPs (2.5 mM, 2 µl), Forward and Reverse primers (10 uM, 1 µl), DNA Template (1 µl) and ddH_2_O (14.75 µl). The amplification procedure consisted of initial denaturation at 98°C for 5 min, degeneration at 98°C for 30 s with 25 cycles, annealing at 53°C for 30 s, extension at 72°C for 45 s, and a final extension at 72°C for 5 min. PCR amplicons were purified and quantified using Vazyme VAHTSTM DNA Clean Beads (Vazyme, Nanjing, China) and a Quant-iT PicoGreen dsDNA Assay Kit (Invitrogen, Carlsbad, CA, USA), respectively.

### 2.3 Analysis of sequencing data

The QIIME2 (v2023.9.1, https://qiime2.org) pipeline and various built-in plugins were used to perform bioinformatic analysis of the sequencing data (Bolyen et al., 2019). The demux plugin, cutadapt plugin (Martin, 2011), and DADA2 plugin (Callahan et al., 2016) were used to demux sequencing and cut primers from the original data, and carry out quality filtering, denoising, merging, as well as the removal of chimeric sequences. DADA2 was also used to denoise the reads into amplicon sequence variants (ASVs) and obtain a profile of the feature sequence. The ASVs were aligned to construct a phylogeny between ASVs and fasttree2 (Price, 2009). Finally, taxonomy were annotated using the feature-classifier plugin (Price, 2009) based on the Navier Bayes classifier and SILVA 138 database reference sequences (Quast et al., 2012). The PICRUSt2 plugin for the QIIME2 and KEGG Orthology databases was used for further predictive functional analyses (Douglas et al., 2020; Kanehisa and Goto, 2000). STAMP was used to identify differential pathways (Parks and Beiko, 2010).

### 2.4 Statistical analyses

The following analysis was performed using R-studio software (V4.3.1). Briefly, the ‘diversity’ function was used to calculate Shannon index of samples, the ‘vegdist’ function was used to calculate the Bray-Curtis distance between samples, and the ‘adonis’ function was used to implement permutational multivariate analysis of variance (PERMANOVA). The functions are included in the vegan package (2.6-4.1). Visualization and principal component analysis (PCoA) were performed using the ggplot2 package (3.5.0). The Wilcoxon rank-sum test was employed to measure difference for alpha diversity index and relative abundance of taxa. PERMANOVA (999 permutations) was employed to identify significant differences between groups (Li et al., 2022). Student’s t-test was adopt to test for significance of microbial function between the two groups. The random-forest clarification model was based on the randomForest package (v4.7–1.1). Pearson correlation coefficient was used to evaluate the correlation between IGA score and depression and anxiety. A linear regression model was used to predict IGA scores. The functions of correlation coefficient and linear regression model are included in the tidyverse package (2.0.0).

## 3 Results

### 3.1 Human subjects and DNA sequencing and screening

Adolescents (aged 15–18 years) with and without acne were enrolled, with a total of 166 participants. Demographic data and clinical parameters are shown in **Supplementary Table S2**. Among acne subjects, the severity of acne in people with negative emotions (mean ± SEM; IGA 2.675 ± 0.090, 72.5% is IGA of 3) was significantly higher than that in people without negative emotions (mean ± SEM; IGA 1.952 ± 0.136, 35.7% is IGA of 3, **Figure 1B**; **Supplementary Table S2**). There were 38 adolescents with depression (mild 13 cases, moderate 14 cases, severe 11 cases) and 44 adolescents with anxiety (mild 19 cases, moderate 9 cases, severe 16 cases). The Pearson correlation coefficient revealed that the correlation between depression and IGA score was 0.1609 (p < 0.05), the correlation between anxiety and IGA score was 0.3191 (p < 0.001, **Supplementary Table S3**). The regression model further supports these findings, with an overall significant fit (p < 0.001, **Supplementary Table S3**). Approximately 14.51% of the variance in IGA Score can be explained by depression and anxiety. Both anxiety (β = 0.4750, p < 1.46E-06) and depression (β = 0.3056, p = 0.00156) were found to be significant predictors of IGA Score, with anxiety showing a stronger effect (**Supplementary Table S3**). The 16S rRNA raw sequence data have been deposited in the Genome Sequence Archive under the accession code (GSA: CRA019782). A total of 13,291,498 raw reads were acquired after 16S rRNA sequencing of the 166 samples. The datasets were then subjected to quality filtration procedures, resulting in 11,291,331 clean reads for subsequent analysis. The average number of sequences per sample was 68,020 and 13850 amplicon sequence variants (ASVs) were identified in the skin bacterial communities of the adolescents (**Supplementary Table S4**). Of the 13,850 bacterial ASVs observed across all samples, 13,571 (98.99%) were identified as phyla, 13,548 (97.82%) as classes, 13,433 (96.99%) as orders, 13,148 (94.93%) as families, and 11,966 (86.40%) as genera (**Supplementary Table S5**). The rarefaction curve, produced by the R software, tended to attain a saturation plateau, showing that the microbiota of the 166 samples were large enough to estimate phenotype richness and microbial community diversity (**Figure 1C**). Thus, the results showed that the sequencing data obtained in this study are reasonable and accurate.

### 3.2 The diversity of skin microbiome of adolescents

**Figure 2:**
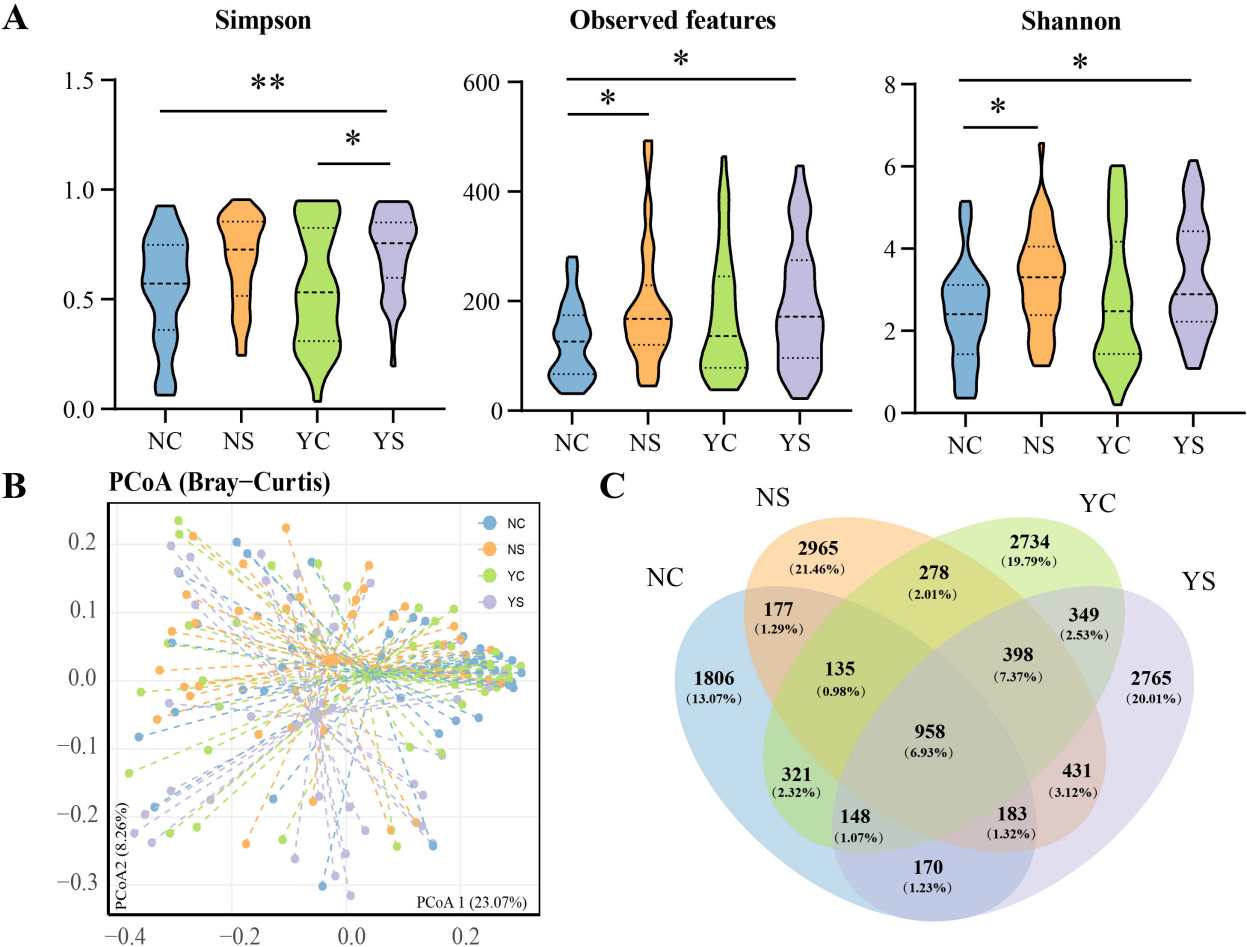
Diversity in adolescents with different characteristics. **(A)** Alpha diversity of four groups. Wilcoxon rank-sum test: *p<0.05; **p<0.01. **(B)** Principal coordinate analyses (PCoA) and permutation multiple variance analysis (PERMANOVA) show the structural differences in the communities of skin bacteria. **(C)** Distribution of amplicon sequence variants (ASVs) across different groups.

We assessed the changes in facial microbiome diversity. To assess the α-diversity, indices for Shannon, Simpson and Observed features were calculated. The indices change across the patient groups, the three indices in YS was significantly higher than in the NC group (**Figure 2A**). Shannon, Simpson, and Observed features in the acne group were higher than those in the non-acne group, regardless of negative emotions. To visualize the structural characteristics of the skin bacterial communities among the different groups, principal coordinate analysis (PCoA) based on Bray-Curtis distances was performed. The PCoA results indicated that axes 1 and 2 accounted for 23.07% and 8.26% of the total variation, respectively. The four groups formed clusters with partial overlap as observed in the plot (**Figure 2B**). Further analysis using permutation multiple variance analysis (PERMANOVA) showed that the facial microbiota composition of adolescents exhibited significant differences (*p*<0.05, **Figure 2B**).

### 3.3 Composition in skin microbiome structure of adolescents

**Figure 3:**
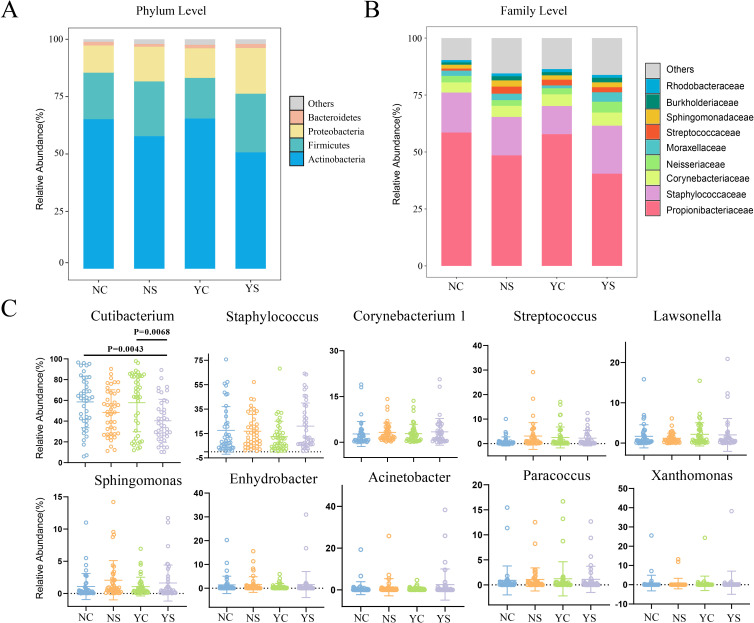
Community relative abundance in different characteristics of adolescents. **(A, B)** Community composition of the skin microbiota among four groups of adolescents at the phylum and family levels, respectively. **(C)** The top ten genera in the skin of adolescents.

Next, we visualized the microbial composition of the facial microbiota. At the phylum level, 28 taxa were observed in all samples, of which 4 different taxa were most common (relative abundance >1%). Actinobacteria (50.75-65.47%), Firmicutes (17.70-25.53%), Proteobacteria (11.84-19.91%), and Bacteroidetes (1.19-1.79%) were the first, second, third and fourth most dominant phyla, respectively (**Figure 3A**; **Supplementary Tables S5**, **S6**). At the family level, 345 taxa were detected in all samples, of which the top 5 for average relative abundance were Propionibacteriaceae (40.49-58.57%), Staphylococcaceae (12.37-21.08%), Corynebacteriaceae (4.45-5.76%), Neisseriaceae (2.50-4.75%), and Moraxellaceae (1.23-4.14%, **Figure 3B**; **Supplementary Tables S5**, **S6**). At the genus level, 972 taxa were detected in all samples, of which the top 10 for average relative abundance were *Cutibacterium* (40.35%-58.53%), *Staphylococcus* (12.36%-21.06%), *Corynebacterium 1* (2.72%-3.41%), *Streptococcus* (0.90%-3.09%), *Lawsonella* (1.19%-2.17%), *Sphingomonas* (1.07%-2.06%), *Enhydrobacter* (0.74%-1.55%), *Acinetobacter* (0.42%-2.53%), *Paracoccus* (0.90%-1.23%), *Xanthomonas* (0.61%-1.07%, **Figure 3C**; **Supplementary Tables S5**, **S6**). Among the top 10 genera, *Cutibacterium* evidently decreased in the YS (*p* < 0.01, **Figure 3C**).

### 3.4 Unique, shared, and core ASVs in skin microbiota of four groups

To investigate the distribution of skin microbiota in the different groups, a Venn diagram was used to analyze the common, unique, and core ASVs (**Figure 2C**). Each group had a unique ASV: NS (2965, 21.46%), YC (2734, 19.79%), YS (2765, 20.01%), and NC (1806, 13.07%). Adolescents with negative emotions from the two groups shared 1853 ASVs, and adolescents without negative emotions shared 1453. Excluding the influence of emotion, 1970 ASVs were shared between adolescents with acne and 1562 ASVs were shared between those with healthy skin. The concept of “core microbiota” is used to identify and describe key microorganisms that are stable and permanent in a microbial community (Astudillo-Garcia et al., 2017). Here, 958 ASVs were shared by all groups. These ASVs primarily belonged to the phyla Proteobacteria (356), Actinobacteria (221), and Firmicutes (203), or the families Corynebacteriaceae (62), Staphylococcaceae (51), and Burkholderiaceae (49).

### 3.5 Skin microbiota as biomarkers for emotion and acne status

**Figure 4:**
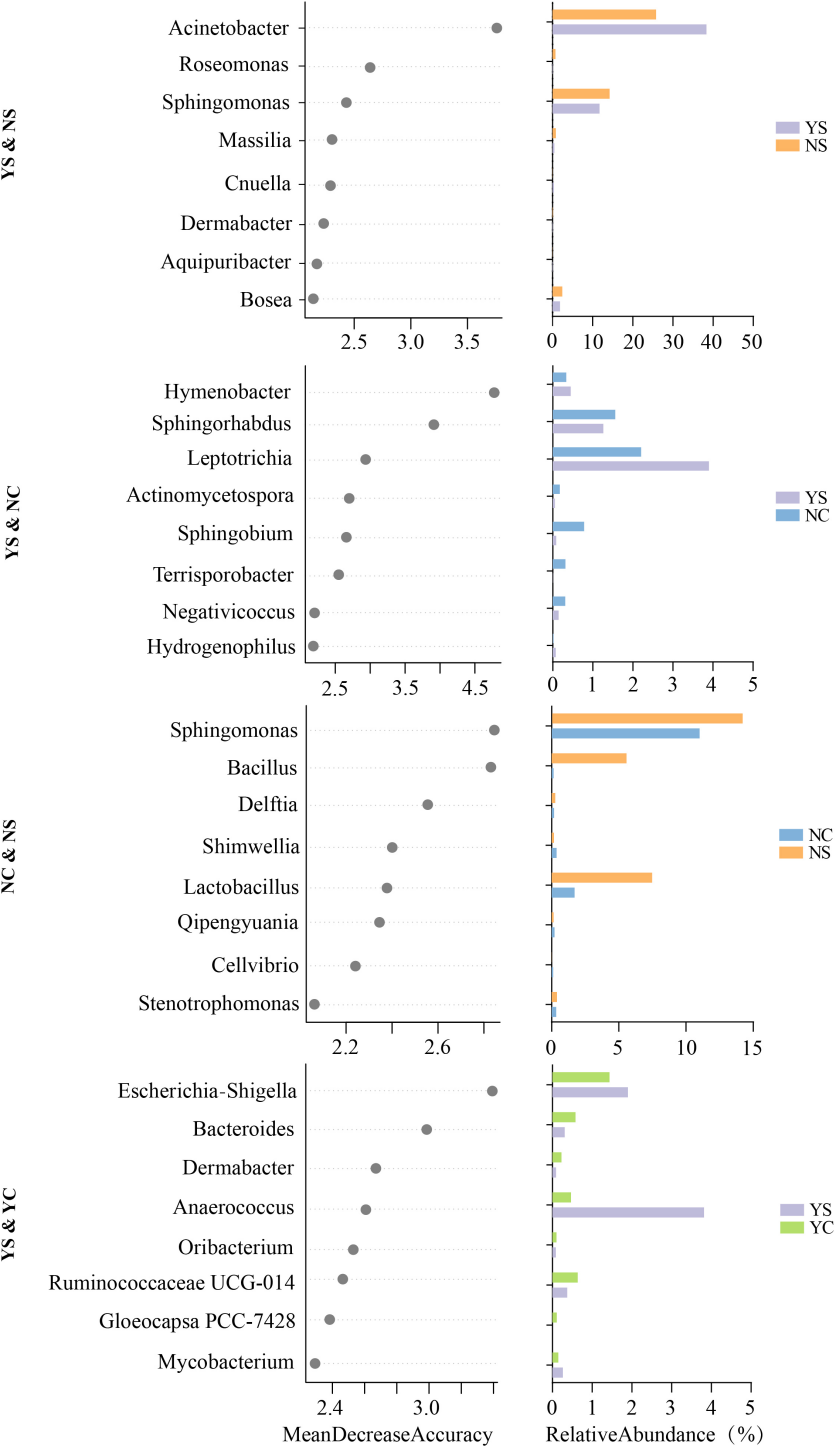
Random forest based on machine learning to explore biomarker of genera between each group pair. Bar plot showed relative abundance of biomarkers in groups.

To determine whether members of skin microbiota can be used as biomarkers to differentiate between emotional and acne status, we established models using the machine learning random forest approach to correlate the emotions and acne status of adolescents with genus-level skin microbiota data. We performed a five-fold cross-validation with five repeats to evaluate the importance of the indicator bacterial genera (**Supplementary Figure S1**). This method has been recognized and applied by other researchers (Zhang et al., 2019). Thus, we defined the top eight genera as biomarkers in the model for all group pairs in the order of group-discriminatory importance (Mean Decrease Accuracy, MDA) (**Figure 4**). As shown in **Figure 4**, *Acinetobacter*, *Roseomonas*, *Sphingomonas*, *Massilia*, *Cnuella*, *Dermabacter*, *Aquipuribacter*, *Bosea* differentiated YS from NS. *Hymenobacter*, *Sphingorhabdus*, *Leptotrichia*, *Actinomycetospora*, *Sphingobium*, *Terrisporobacter*, *Negativicoccus*, *Hydrogenophilus* differentiated YS from NC. *Sphingomonas*, *Bacillus*, *Delftia*, *Shimwellia*, *Lactobacillus*, *Qipengyuania*, *Cellvibrio*, *Stenotrophomonas* differentiated NC from NS. *Escherichia-Shigella*, *Bacteroides*, *Dermabacter*, *Anaerococcus*, *Oribacterium*, *Ruminococcaceae UCG-014*, *Gloeocapsa PCC-7428*, *Mycobacterium* were the most important genera for discriminating the acne status (differentiating YS from YC).

### 3.6 Functional predictions of facial microbiota

**Figure 5:**
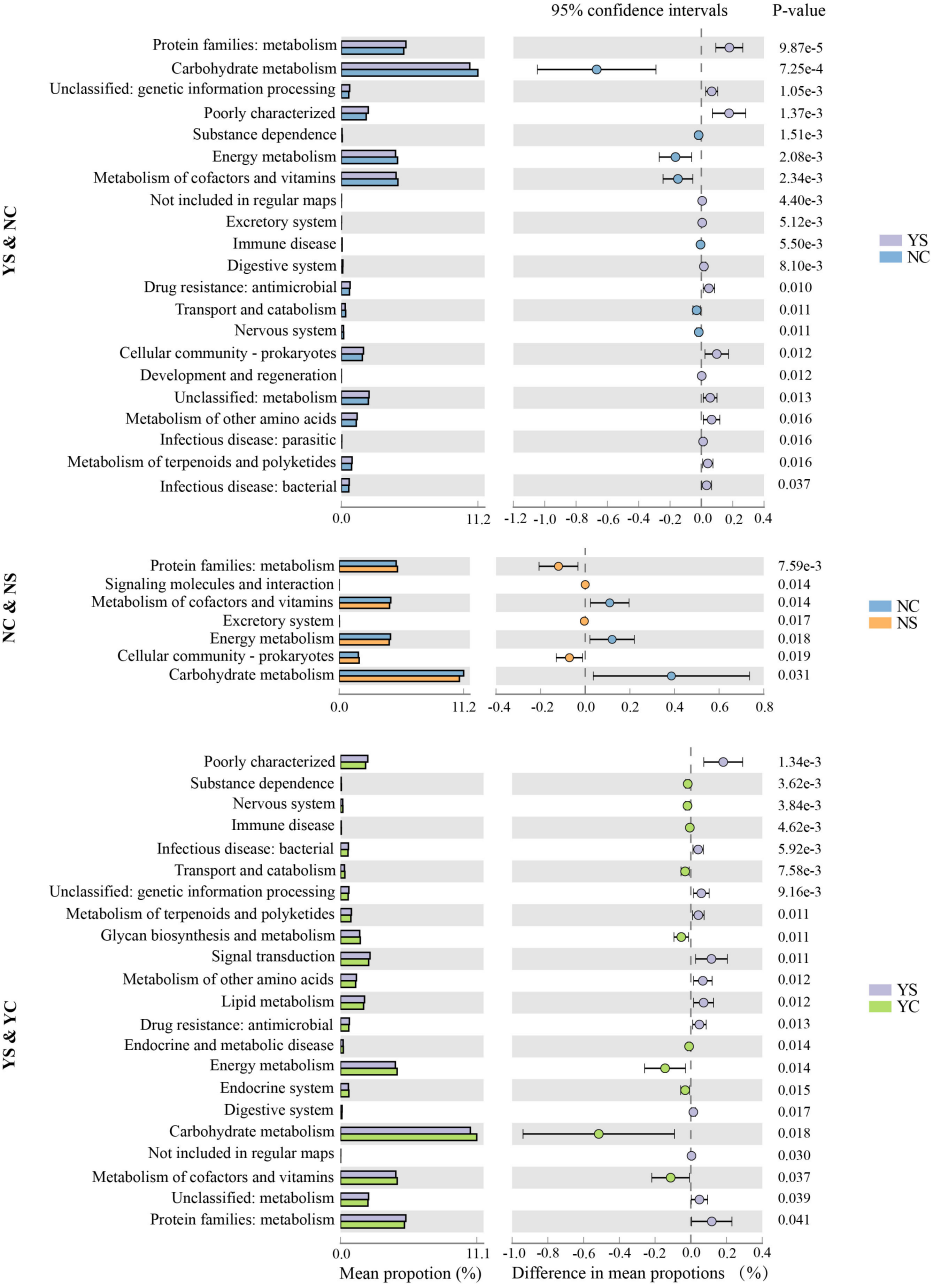
Several functions were detected existing significant difference in groups.

To further understand the biological functions of the microbial community, metagenomic functions of the bacteria were predicted using the PICRUSt2 pipeline. A total of 7753 predicted metagenomic functions were obtained and annotated using KEGG Orthology (KO, **Supplementary Table S7**). KO exists primarily in metabolism, organismal systems, human diseases, and cellular processes. Various functional pathways of microbiota were observed in the different groups, as shown in the heatmap (**Supplementary Figure S2**), suggesting discrepant microbial functional potential among the microbiota of several groups. In the YS & NC and NS & NC groups, 21 and 7 pathways differed significantly (*p* < 0.05), respectively; these were significantly higher than the differences observed in the other two comparisons (**Figure 5**). Compared with the NC group, pathways were more abundant in the YS group, including protein families: metabolism, Digestive system, Drug resistance: antimicro, and the excretory system. Compared with NC, protein families metabolism, signaling molecules and interaction, excretory system, cellular community, and prokaryotes showed a preference for NS (*p* < 0.05). In addition, 22 pathways were found to differ significantly (*p* < 0.05) between YS and YC.

## 4 Discussion

Negative emotions and skin problems such as acne are two important characteristics of adolescents; however, the relationship between the skin microbiome, mood, and acne remains unclear. Therefore, a better understanding of the skin microbiome composition of adolescents with acne and negative emotions will be helpful for managing the facial microbiome of specific populations, by providing evidence to support targeted acne treatment in the future. Focusing on negative emotions and acne, this study characterized the composition and diversity patterns of facial skin microbes in adolescents with and without negative emotions and acne, compared the differences, and detected important bacterial genera that could distinguish different groups. In addition, the metagenomic function of the skin microbiota of adolescents was predicted, and functional differences between the groups were compared. Research suggests that negative emotions aggravate acne in adolescents, and this process may be related to the skin microbiota.

The results of this study are consistent with those of previous reviews, adolescents with negative emotions have more severe acne (Cunliffe, 1980). The forehead was selected as the skin sampling point to eliminate the influence caused by different skin sites (Sun et al., 2024). Completely opposite results regarding the difference in skin microbial diversity between patients with and without acne have been reported. There are two reports investigating the skin microbiota of acne patients in China, showing that the α-diversity of skin microbiota in the acne group is greater than that in the healthy control group, and there is a difference in β-diversity, which is consistent with this study (Li et al., 2019; Shi et al., 2021). However, there is also evidence showed that patients with acne have significantly reduced alpha diversity in their skin microbiota (Cavallo et al., 2022). There are even studies that show no difference in the diversity of skin microbiota between an acne group and a healthy group has also been reported (Kim et al., 2021). Shannon, Simpson, and observed features indexes were used to evaluate the α-diversity of facial microbiota of adolescents. The results showed that the α-diversity in the acne group was higher than that of the healthy group with or without negative emotions, although not all differences were significant, and YS was significantly higher than NC in three indexes. In some cases, individuals with acne may exhibit a higher microbial richness and diversity than healthy individuals (Huang et al., 2023). Negative emotions can exacerbate skin inflammation and disrupt skin balance, making the skin more susceptible to environmental influences, allowing more microbial colonization and increasing the abundance of harmful bacteria (Sandoval and Ayres, 2017). In β-diversity, significant differences were observed among the four groups. Compared to the other groups, negative emotions had minimal effects on the facial microbiome of adolescents with healthy skin but increased facial microbiome differences between people with acne. Compared with healthy people, people with acne generally have a more unstable microbiota structure and show higher sensitivity to negative factors, such as negative emotions (Yang et al., 2022). Interestingly, YS had a completely different microbial community structure than NS, which may be related to more severe acne in people with negative emotions. The IGA scores and diversity results suggest that the facial microbiome of adolescents with negative emotions and acne deserves further attention. The key reason for the different results in the literature may be difference in origin of the subjects, and the different backgrounds of the subjects, such as work, lifestyle, and age, may have increased sample diversity. In addition, operational differences may have affected the results. Therefore, in the future, a more detailed record of the various factors that may affect skin microorganisms should be made during the research implementation to provide more references for subsequent researchers.

The species composition results in the present study were consistent with those of other studies on facial skin microbes (Filaire et al., 2019; O’Sullivan et al., 2019; Yamazaki et al., 2017). Actinobacteria, Firmicutes, Proteobacteria, and Bacteroidetes were the predominant phyla in the skin of the adolescent population, accounting for at least 97.6% of the total abundance in all samples (**Figure 3A**; **Supplementary Table S5**). Considering the present results and those from previous studies leads to the conclusion that, no matter what factors affect the skin, these bacteria are core components of the skin microbial community (Filaire et al., 2019). At the genus level, *Cutibacterium* was the most abundant bacterium in the facial skin, which may be because the sample site in this study was the forehead. The forehead is one of the most densely populated parts of the facial sebaceous glands and is suitable for *Cutibacterium* to survive and breed (Spittaels et al., 2020). The acne group had a low abundance of *Cutibacterium*, whereas the adolescents with negative emotions and acne had the lowest abundance of *Cutibacterium*. *Cutibacterium* comprises a variety of harmful and beneficial strains that carry different virulence genes. *C. acnes*, for example, is thought to be significantly associated with the development of acne, but whether it plays a positive or negative role remains debatable (Dreno et al., 2017). *Staphylococcus* and *Corynebacterium 1* were the second and third most abundant bacteria on teenagers’ faces, respectively, perhaps because the study was conducted in southern China where a humid environment is more suitable for colonization by *Staphylococcus* and *Corynebacterium 1* (Grice et al., 2009; Grice and Segre, 2011). Notably, the presence of *Bacillus*, a genus known to form symbiotic relationships with *Demodex*, has been observed in the skin microbiome of adolescents. It has been documented that the *Demodex*-associated bacterial proteins were implicated in the inflammation induction (Kubiak et al., 2018). The species composition analysis suggest that the facial community structure of adolescents with negative emotional acne should be detailed in the future, especially *Cutibacterium*. This group were not only likely to have more severe acne, but may also have new mechanisms for the development of acne. Simultaneously, the presence of *Bacillus* suggests that greater attention should be directed toward *Demodex* in future studies. Considering the host-endosymbiotic relationship and its interactions may provide valuable insights into the pathogenesis of acne.

Individual characteristics of the skin microbiome are often driven by low-abundance species that are critical for maintaining physiological functions of the skin (Fierer et al., 2010). Therefore, we selected random forest, an analytical method that has advantages for analyzing low-abundance microorganisms, to mine biomarkers (Ren et al., 2019). Completely different biomarkers were observed in the group comparison. A Venn diagram also showed that at least 14.07% of the ASVs in the different groups were group-specific microorganisms (**Figure 2C**), indicating significant differences in facial skin microbial characteristics among the four groups. Compared with the NS, there was a higher abundance *Acinetobacter* and a lower abundance *Roseomonas* in the YS. *Acinetobacter* is an important pathogen in skin diseases and is widely present in infected or wounded skin (Loomis et al., 2021). Interactions between *Acinetobacter* and other microorganisms (*C. acnes* or *Staphylococcus*) may further complicate the skin immune response (Lee et al., 2019). *Roseomonas* is a beneficial bacterium that regulates the immune response and supports the skin barrier function, which can maintain skin health (Ito and Amagai, 2022). *Roseomonas mucosa*, in particular, has been used to treat conditions such as atopic dermatitis (Myles et al., 2018). Negative emotions can lead to more severe acne (**Figure 1B**; **Supplementary Table S3**), when acne is accompanied by negative emotions, the bacterial biomarkers of the disease change. *Acinetobacter* and *Roseomonas* may play an important role in the imbalance of the skin microbiome caused by negative emotions and the inflammatory induction of more severe acne. *Hymenobacter*, *Sphingorhabdus*, *Leptotrichia* and five genera were identified as biomarkers for comparing YS and NC. In addition, eight HP&YP biomarkers were identified in YS&YC and NS&NC to distinguish the different groups. In the future, more methods should be developed to identify key microorganisms and combine the various characteristics of clinical acne. If the mechanism underlying acne is not fully understood, this practice can provide more directions for relevant in-depth research.

Changes in the composition of the skin microbiota lead to changes in the function of the skin microbiota, which inevitably affect the occurrence and development of skin diseases (Guo et al., 2023). A total of 7,753 predicted biological functions were obtained in this study, and the characteristics of the function in skin microbiota changed in different groups, as shown in the heat map. The primary changes in NS and NC in the KEGG pathway involved the following biological processes: energy metabolism, signaling molecules, and interactions, which is consistent with a previous study in healthy rats with acne (Zhu et al., 2022). Compared with NC, YS had more differential metabolic pathways than NS, indicating a more complex microbial metabolism in YS facial microorganisms. In addition to most of the differential metabolic pathways that contain NS&NC, differences in the metabolism of amino acids between YS and NC were also observed. Alterations in these substances may significantly disrupt host skin homeostasis by stimulating skin keratinocytes and immune cells (Chen et al., 2018). This may explain why YS acne is more severe than NS acne. In addition, some comparisons did not reveal significant differences in metabolic pathways, such as YS&NS. This may be because the prediction was incorrect, certain metabolic pathways were masked, or the study sample did not reflect the true level of metabolism.

Although potential associations exist between acne, negative emotions, and the skin microbiome, this study has several limitations. First, unexamined factors, such as skin care (including the frequency of facial cleansing, the type of cleansers used, and traditional therapies for acne etc.) and diet (including different glucemic index foods etc.), may influence acne severity, negative emotions, and the skin microbiome. Second, individuals experiencing negative emotion states may exhibit lower adherence to skincare routines, potentially affecting the skin microbiome. Therefore, it is necessary to expand the data further to investigate the association and causality between adolescent acne and negative emotions, as well as to examine the role of skin microbes in this relationship. Providing more data on dermatological, psychological, and microbial characteristics in adolescents is crucial for advancing research in this field.

## 5 Conclusion

This study explored the association between skin microbiome composition, the presence of negative emotions, and acne in an adolescent cohort. The results suggest that individuals experiencing both acne and negative emotions possess a unique skin microbiome profile, which potentially contributes to the heightened severity of acne observed in this group. Notably, *Cutibacterium*, *Acinetobacter*, and *Roseomonas* have been identified as key microbial taxa that may play a role in the pathogenesis of acne linked to emotional stress. Therefore, uniform management of adolescent acne problems may be inappropriate. The skin microbiome of adolescents with negative emotions deserve more detailed attention. Interventions tailored to the microbial characteristics of this population, such as the application of probiotics, prebiotics, and customized skincare regimens, could help alleviate acne severity triggered by emotional stress while promoting skin microbiome homeostasis. However, the use of 16S rRNA gene sequencing did not allow species-level biomarker identification, highlighting the need for future research to employ metagenomic and metabolomic approaches to uncover specific biomarkers. Further investigations of *Cutibacterium*, *Acinetobacter*, and *Roseomonas* are recommended to identify probiotic candidates or pathogenic bacteria, ultimately providing a scientific basis for the development of personalized microbiome-focused acne therapies.

## Data Availability

The 16S rRNA raw sequence data have been deposited in the Genome Sequence Archive under the accession code (GSA: CRA019782).

## Supplementary material

Supplementary Figure 1 Five-fold cross-validation with five repeats were used to evaluate the importance of indicator bacterial genera, including YS versus NS, YS versus NC, NC versus NS, YS versus YC.

Supplementary Figure 2 KEGG metagenomic function of bacteria was predicted using the PICRUSt2 pipeline. The heatmap display abundance of function at KEGG level B. The value represented the normalization of functional abundance in this dataset, which higher numbers indicated greater relative abundances with colors ranging from dark red to blue.

Supplementary Table 1 IGA Scale: Adapted from the FDA center for drug evaluation and research on acne vulgaris.

Supplementary Table 2 Human demographics.

Supplementary Table 3 Correlation analysis between negative emotion and severity of acne.

Supplementary Table 4 Number of sequence of ASVs in samples.

Supplementary Table 5 Taxonomy information of ASVs.

Supplementary Table 6 Proportion of bacterial abundance of four groups, including top 5 phylums, top 10 families and top 20 genus.

Supplementary Table 7 The abundance of KO in each sample predicted by PICRUSt2.
