# Supplementary figures and images for: Amplicon-based analysis reveals link between adolescent acne and altered facial skin microbiome induced by negative emotional states
Source: Front Cell Infect Microbiol. 2025 Mar 19;15:1543616. doi: 10.3389/fcimb.2025.1543616 (PMC11961944; doi:10.3389/fcimb.2025.1543616)

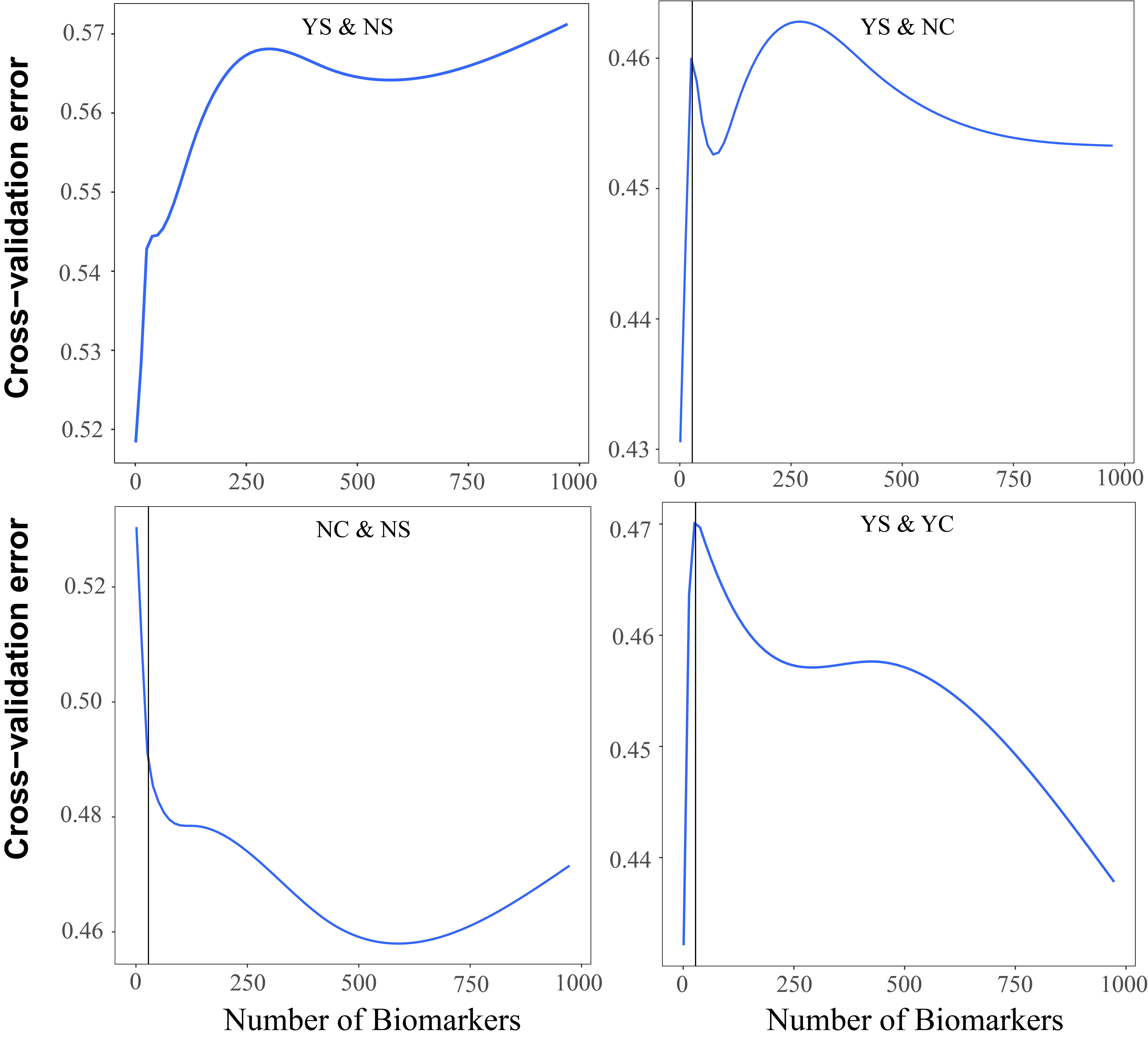

Supplement: Supplementary Figure 1 — Five-fold cross-validation with five repeats were used to evaluate the importance of indicator bacterial genera, including YS versus NS, YS versus NC, NC versus NS, YS versus YC. [file Image1.jpeg]

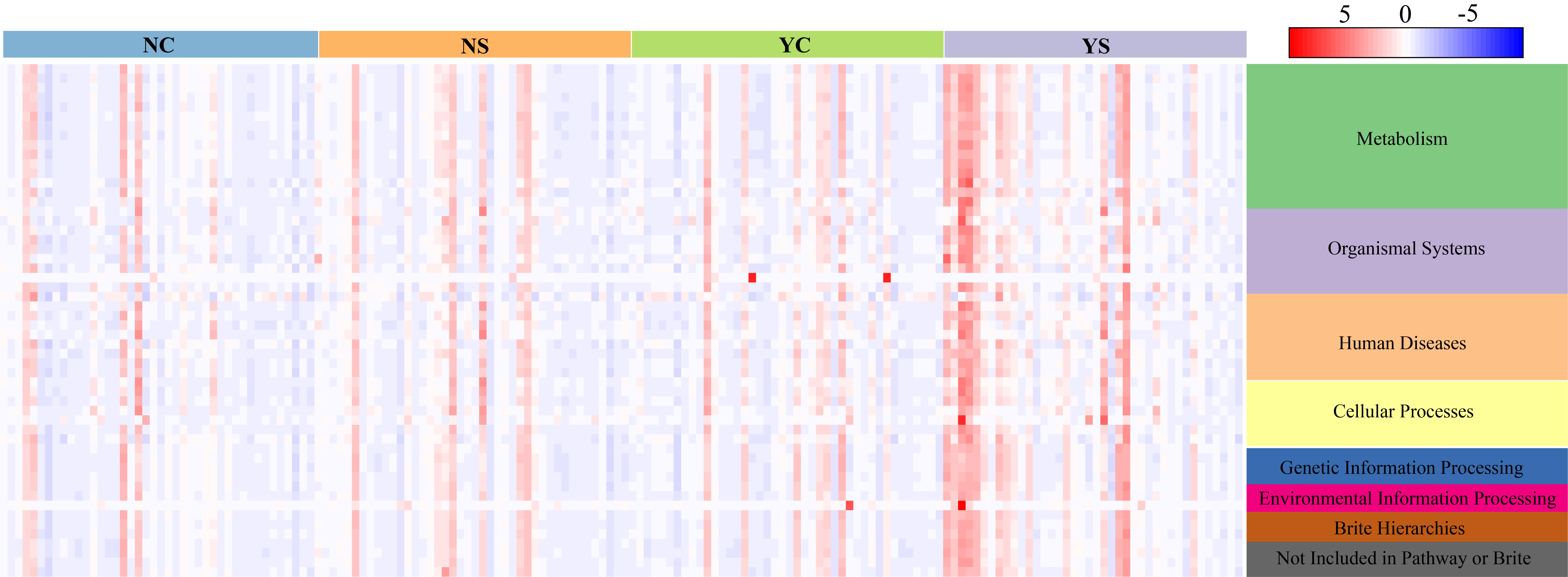

Supplement: Supplementary Figure 2 — KEGG metagenomic function of bacteria was predicted using the PICRUSt2 pipeline. The heatmap display abundance of function at KEGG level B. The value represented the normalization of functional abundance in this dataset, which higher numbers indicated greater relative abundances with colors ranging from dark red to blue. [file Image2.jpeg]
